# Genetic diversity and molecular evolution of sugarcane mosaic virus, comparing whole genome and coat protein sequence phylogenies

**DOI:** 10.1007/s00705-022-05572-x

**Published:** 2022-08-23

**Authors:** Khalid Muhammad, Venura Herath, Khadija Ahmed, Muhammad Tahir, Jeanmarie Verchot

**Affiliations:** 1 Atta-ur-Rahman School of Applied Biosciences, National University of Sciences and Technology, Islamabad, Pakistan; 2 Department of Plant Pathology and Microbiology, Texas A&M University, College Station, TX 77843 USA; 3 Department of Agriculture Biology, Faculty of Agriculture, University of Peradeniya, 20400 Peradeniya, Sri Lanka

## Abstract

Sugarcane mosaic virus (SCMV) is a widely distributed potyvirus that causes mosaic disease in sugarcane, maize, sorghum, canna, and other monocot species worldwide. This study used 139 SCMV full-length genome sequences to analyze the phylogenetic relatedness of geographically distinct isolates. The phylogenetic analysis revealed four major groups of SCMV isolates that relate to their primary host. The geographic locations for some isolates appear to be mismatched within the tree, suggesting either that convergent molecular evolution has occurred or that the tree reconstruction produces statistically significant incongruences that create uncertainty in the true evolutionary relationships of these virus isolates. Recombination analysis showed hot spots across most of the genome except in the coat protein (CP) coding region. We examined 161 SCMV CP sequences from the GenBank database, including sequences from samples collected in Pakistan, a region that has not been included in prior phylogenetic studies. These data suggest that the SCMV isolates from sugarcane (*Saccharum officinarum*) predate isolates from all other hosts, regardless of their geographic origins.

**Supplementary Information:**

The online version contains supplementary material available at 10.1007/s00705-022-05572-x.

## Introduction

The family *Potyviridae* currently comprises more than 30% of plant virus species, and the genus *Potyvirus* includes the largest number of species within the family [1]. The global distribution of these viruses is attributed to agricultural trade, especially for seed-borne viruses, as well as dispersal by migrating arthropod vectors. Trade and travel have enabled the establishment of potyviruses in different regions of the world and the introduction into other crops [2, 3]. According to Gibbs et al. [4], speciation among potyviruses results from mutations and selection, which enables them to become established in weeds or crops once they are introduced into new regions. Studies have indicated that recombination is not a common mechanism for potyvirus speciation, and this makes phylogenetic trees informative for investigating the evolutionary origins of various potyviruses. Many lineages are defined by the relationships of their recognized hosts, which are grouped at the order level [4]. Phylogenetic investigations have identified eleven basal lineages of potyviruses, which are named according to the earliest-described species member [4–6].

The major members of the sugarcane mosaic virus group are sugarcane mosaic virus (SCMV), johnsongrass mosaic virus (JGMV), canna yellow streak virus (CaYSV), cocksfoot streak virus (CfSV), and pennisetum mosaic virus (PeMV) [7–9]. Researchers typically view the sugarcane mosaic virus group as viruses from Old World grasses; however, the inclusion of CaYSV, whose host range includes canna, achira, common bean, and chenopodium, within this group, suggests that the SCMV group has a broader host range and cannot be so narrowly defined [9–12]. The global trade of cereals and pulses, as well as ornamental plants, is likely to be a major factor influencing the genetic diversity of viruses within the SCMV group [13].

SCMV was first detected in sugarcane in 1919 and occurs throughout the world, causing mosaic disease in maize, sugarcane, canna, and other gramineous species worldwide [12, 14]. SCMV is transmitted by aphids in nature or by mechanical inoculation. As for all members of the family *Potyviridae*, the genome consists of a positive-sense single-stranded RNA with a genome size of approximately 9–10 kb. The flexuous filamentous virion possesses a genome-linked protein (VPg) at the 5’ end and a poly(A) tail at the 3’ end. The genome encodes a single large polyprotein that is proteolytically processed into functional proteins [5].

Researchers have tried to understand the basis for genetic changes across the complete genome sequences of SCMV isolates obtained from different hosts and geographic locations [7, 15]. The preferential use of identical or synonymous codons (i.e., codons encoding the same amino acid) is a pattern shaped by natural selection, mutation pressure and bottlenecks, viral fitness, replication, protein structure and function, and adaptation to the host and environment [5, 7, 16]. Potyvirus genomes characteristically contain hypervariable regions, such as the 5’ UTR, and show extensive intraspecific recombination, which has been suggested by some researchers to confound phylogenetic studies investigating evolutionary features [5, 7, 16]. Traditional phylogenetic studies examine different genomic regions, which, for potyviruses, may produce different phylogenetic trees that cannot be used to group isolates into strains based on nucleotide or amino acid sequence similarities [7, 15–17].

For many SCMV isolates, the nucleotide sequences of the coat proteins (CPs) have been amplified for diagnostic studies, and these sequences have been deposited in the GenBank database [17–19]. These sequences are often studied to evaluate selection pressure, evolutionary lineages, and phylogenic tree topologies [4, 20]. Sugarcane is a major crop in Pakistan, and most of the cultivated varieties are susceptible to SCMV infection [21]. Diagnostic studies have shown two SCMV strains, A and F, to be prevalent in Pakistan [22]. Early sequence analysis of the CP genes suggested that the SCMV variants in Pakistan represent novel populations [23]. Here, we have evaluated the full-length genome phylogenies to understand SCMV diversity and included SCMV CP sequences from Pakistan [18] to understand their phylogenetic relatedness to global isolates.

## Materials and methods

### Virus sequence retrieval, alignments, and phylogenetic analysis

One hundred thirty-nine full genome, polyprotein, and CP sequences were retrieved from the NCBI (National Center for Biotechnology Information) GenBank database and imported into Geneious Prime v2020.0.1 and MEGA-X v10.1.8 [18, 21, 24]. Infected field samples of sugarcane were collected in Pakistan, and the SCMV CP sequences were amplified by RT-PCR and cloned into the plasmid pTZ57R/T as reported by Akbar et al. [18]. The NCBI nucleotide sequence accession numbers and Protein IDs for the curated genome and CP sequences used in this study are provided in Supplementary Tables S1 and S3. MUSCLE software, built in to MEGA X v10.1.8, was used for nucleotide and amino acid sequence alignments [24, 25]. Phylogenetic analysis was performed by the maximum-likelihood (ML) method, using the entire nucleotide sequence alignments, codon-based alignments, and polyprotein sequence alignments, with 1000 bootstrap replicates. ProTest v3.4.2 was used to determine the best model for nucleotide and codon-based alignments, and the general time-reversible (GTR) substitution, gamma distribution, and proportion of invariable sites (GTR + G + I) model was chosen [26]. iTOL v5.0 and v6.5.2 were used to display and annotate the phylogenetic trees [27]. Sequence similarity network analysis of full genome sequences and CP coding sequences was carried out according to country of origin, using Simplot + + v1.0 [28].

### Analysis of recombination, diversity, and phylodynamics of SCMV isolates

Recombination events were detected using the RDP5 recombination detection program with default settings and a *p*-value cutoff of 0.01. This program employs nine statistical detection methods: RDP, GENECONV, Bootscan, Maxchi, Chimaera, SiScan, Phylpro, LARD, and 3Seq [29–31]. Events that were detectable by at least five of the nine methods were tabulated, and recombination breakpoints were identified manually and tabulated. Recombination breakpoints identified using RDP5 were validated using the genetic algorithm GARD with the following parameter settings: Faster run mode, General Discrete for site-to-site rate variation, and rate classes 3. DnaSP v5 software was used to assess the nucleotide diversity (Pi) along 139 SCMV complete genomes with a sliding window size of 100 bp and a step size of 25 bp [16, 32].

## Results

### Phylogenetic analysis of complete SCMV genome sequences

**Fig. 1:**
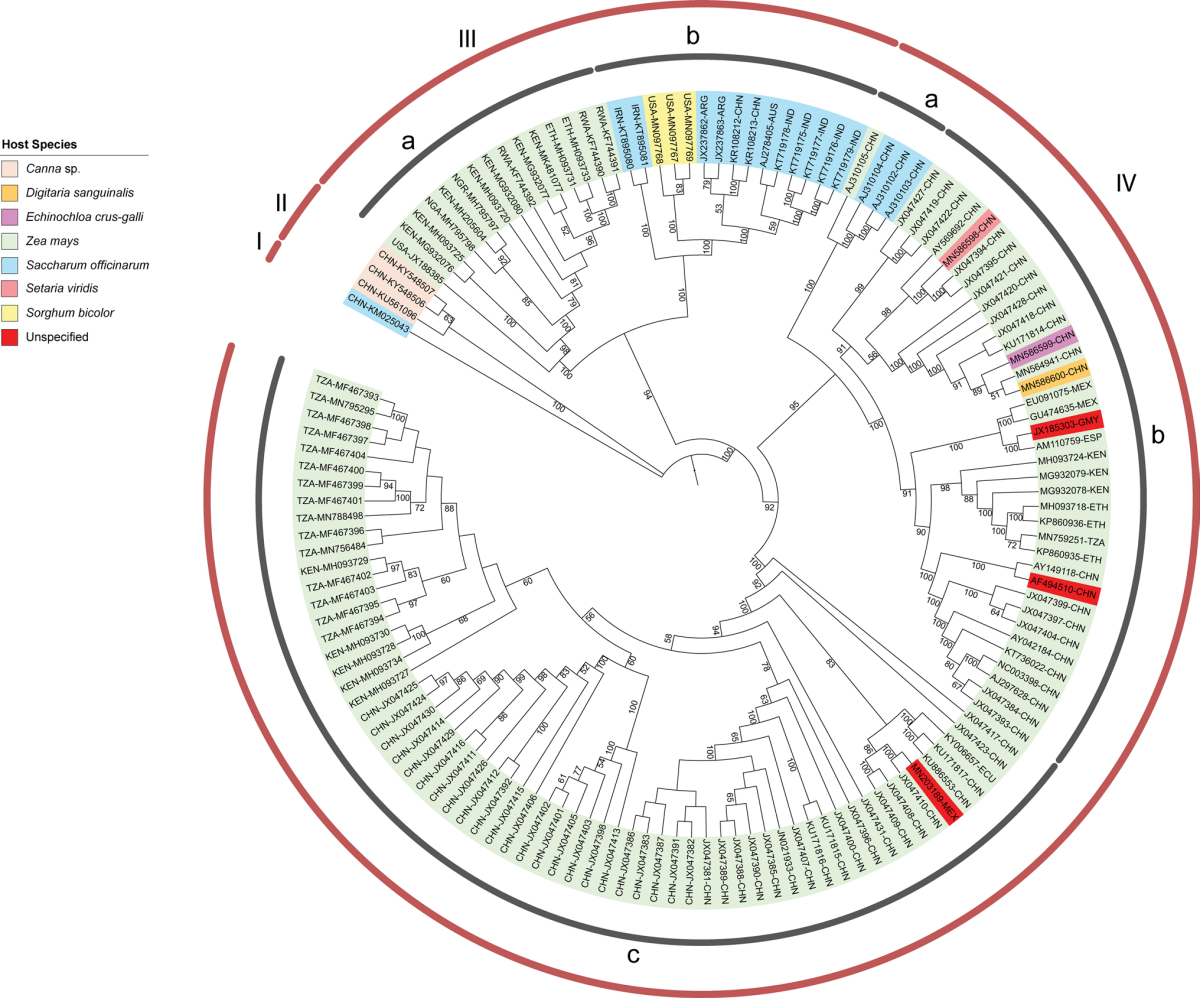
Maximum-likelihood phylogenetic tree based on full-length genome sequences of SCMV isolates. Four lineage groups were identified based on clustering extending from the most distant node. Subgroups a, b, and c extend from a common intermediate branch within the deeply rooted lineage group. All sequences are identified by their GenBank ID and abbreviation indicating the country of origin. The colors identifying the host species are explained in the legend.

**Fig. 2:**
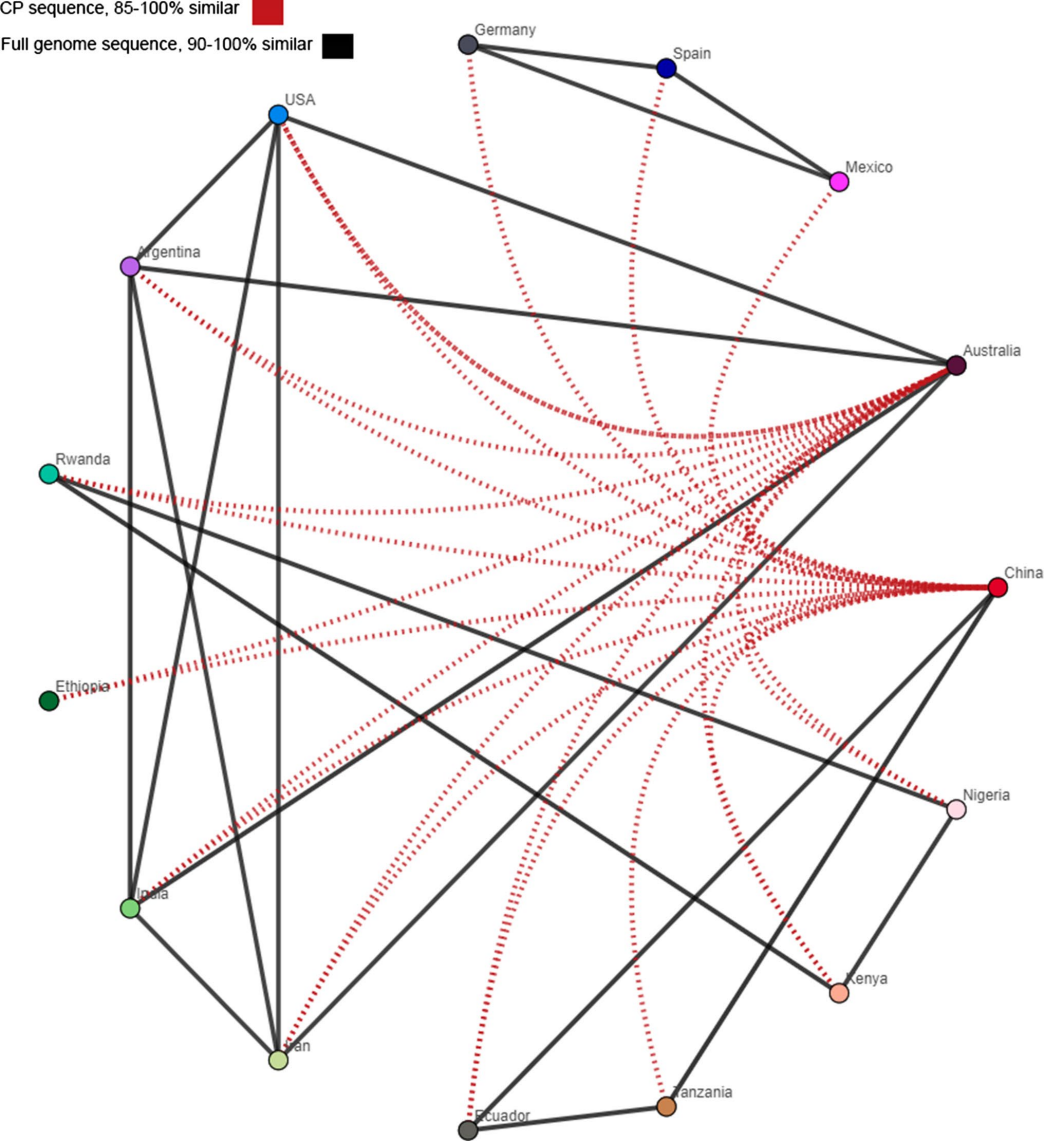
Similarity network analysis of SCMV genome sequences conducted using SimPlot++, based on the country of origin. Black lines indicate sequences with high overall sequence identity (90–100%), and red lines represent identity among the CP coding sequences (85–100%). Thresholds were chosen to best represent how geographically distinct isolates share high sequence similarity.

To investigate the genetic variability within the species *Sugarcane mosaic virus*, we compiled and analyzed 139 previously reported SCMV genome sequences from Europe, North and South America, Asia, and Africa that were available in the GenBank database (Supplementary Table S1). These sequences were deposited and updated between the years 2000 and 2020. Their genome lengths ranged from 9,192 to 9,628 base pairs, and their polyprotein lengths ranged from 3,046 to 3,071 amino acids. The hosts were *Canna* spp., *Digitaria sanguinalis, Echinochloa crus-galli, Saccharum officinarum, Setaria viridis, Sorghum bicolor*, and *Zea mays*, and some hosts were not specified (Supplementary Table S1). We produced one ML phylogenic tree based on the complete nucleotide sequences and another based on the complete protein sequences. A side-by-side comparison of the ML trees was carried out to determine if the tree topologies showed matching visible nodes, branches, and leaf order. The ML tree presented in Fig. 1 has three deeply rooted branches, and one of those branches diverges into two major clusters. The two major clusters were identified as four major groups. Groups I and II include four individual viruses that infect sugarcane and *Canna* spp. Group III has two clusters. Group IIIa consists of maize-infecting viruses from East Africa and the USA. Group IIIb consists of sugarcane- and sorghum-infecting isolates from Iran, the USA, Argentina, Australia, and India. Group IV is comprised mostly of isolates infecting maize in China but also includes isolates from sorghum, identified weeds (*D. sanguinalis*, *E. crus-galli*), and unspecified hosts. The geographic locations for these isolates appear to be mismatched within the tree, suggesting either that convergent molecular evolution has occurred or that the tree reconstruction produces statistically significant incongruences that do not lead to a high-confidence model [33]. The group and subgroup assignments highlight the clustering of isolates according to their hosts. Notably, virus isolates cluster into subgroups that may have different geographic origins. Isolates were compared across wide geographic areas using Simplot ++. Using a similarity threshold of 90% for the entire genome or 85% for the coat protein coding region, we observed that geographically distinct isolates shared extensive sequence similarity (Fig. 2). These data indicate that the phylogenetic subgroups are more likely to be defined by host than by geography.

### Analysis of recombination and variability across the genome of SCMV isolates

Recombination analysis was carried out using 139 full-length genome sequences. Recombination breakpoints identified using RDP5 were confirmed using the genetic algorithm for recombination detection (GARD), with a general discrete distribution of sites into three rate classes [34]. The outputs of both detection programs confirmed 38 common recombination events with highly significant *p*-values. We tabulated the major breakpoints in each gene (Supplementary Table S2). Notably, the SCMV genomes assigned to phylogenetic groups I and II did not appear to be recombinants. Group IIIa had four genomes that appeared to be recombinants, and group IIIb had five recombinant genomes. One sequence was assigned to group IVA, and the majority of recombinant sequences, a total of 47 sequences, were assigned to phylogenetic group IVb. Eleven recombinant sequences were in group IVc (Supplementary Table S2).

**Fig. 3:**
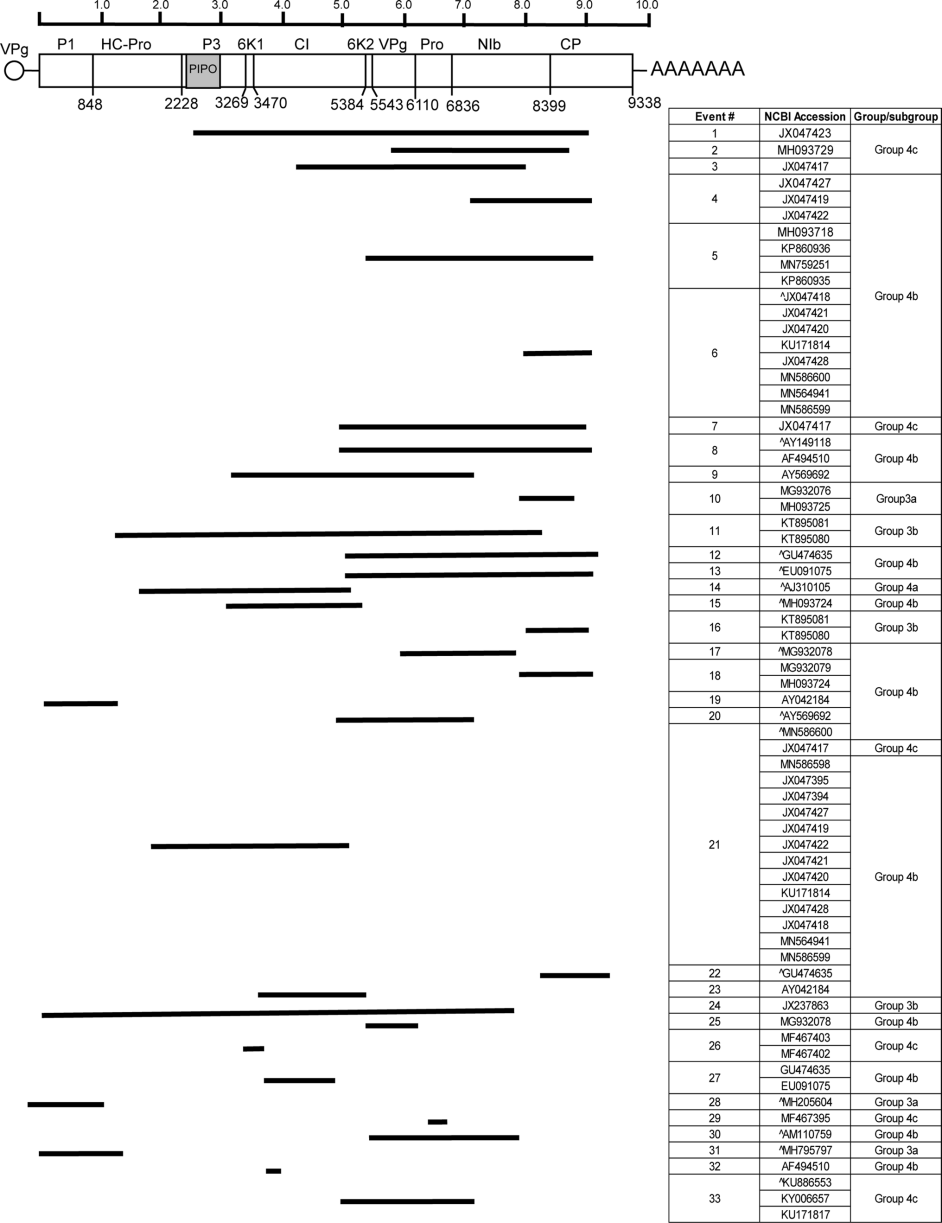
Recombination analysis of SCMV genome sequences. The diagram highlights segments extending between major recombination breakpoints for each virus isolate. The NCBI accession numbers and phylogeny group/subgroups are identified on the left. The genome encoding the polyprotein is at the top, and the amino acid positions for the proteinase cleavage sites are identified below the genome map. The viral genome-liked protein (VPg) is at the 5’ end, and the polyA tail is at the 3’ end of the genome. The mature proteins are identified above the open box: P1 proteinase (P1), helper component proteinase (HC-Pro), pretty interesting *Potyviridae* ORF (PIPO), P3, 6K1, cylindrical inclusion (CI) protein, 6K2, viral protein genome linked (VPg), protease (Pro), nuclear inclusion b (NIb), and coat protein (CP).

Next, we mapped the recombination breakpoints across the SCMV genome. The SCMV genome, as for all potyviruses, encodes a long polyprotein that is proteolytically cleaved by viral proteinases to produce mature products. These proteins, in linear order, are named P1, helper component proteinase (HC-Pro), P3, 6K1, cylindrical inclusion (CI), 6K2, VPg, nuclear inclusion protein a-protease (Nia-Pro), nuclear inclusion protein b (Nib), and coat protein (CP). There is a short open reading frame (ORF) overlapping the P3 ORF that encodes a protein named PIPO [35]. Interestingly, the majority of recombination breakpoints were within the NIb-CP region of the polyprotein, between nucleotide (nt) positions 6837 and 9338 (Fig. 3, Supplementary Table S2). The second most common location for recombination breakpoints was the central region of the genome, stretching from P3 to Nia-Pro or the 5’ end of the Nib coding region. Few genomes had breakpoints in the P1 or HC-Pro coding region (Fig. 3, Supplementary Table S2). Except for subgroup IVb, the majority of isolates showed a pattern of breakpoints that is similar among the subgroup members.

**Fig. 4:**
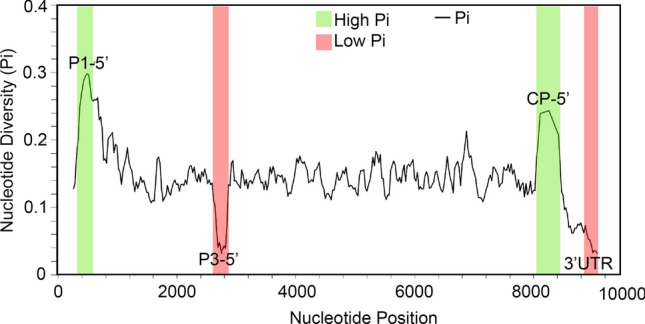
Nucleotide diversity analysis of 139 SCMV genome sequences with a window size of 100 bp and step size of 25 bp. The graph shows the nucleotide diversity across the viral genome. Pi is the average number of nucleotide differences per site between two sequences. Highly polymorphic sequences are identified by pink boxes, and the most conserved regions are identified by green boxes.

Potyviruses are well known for having hypervariable regions [15]. Prior studies of SCMV reported nucleotide sequence variation and polyprotein variation in a subset of isolates [16]. Here, we used DnaSP v5 software to assess the nucleotide diversity (Pi) across the full-length SCMV genome, using a window size of 100 bp and step size of 25 bp [32]. The total number of mutations was 6930 across 4427 sites, and the average number of nucleotide differences between sequences (*k*) was 1227.3. The nucleotide diversity (*Pi*) across the genome was 0.14, similar to what has been reported previously [16], showing that the 5’ end of the P1 and coat protein (CP) coding sequences are highly polymorphic (Fig. 4).

### ML phylogeny of the SCMV CP ORF shows clustering according to host

**Fig. 5:**
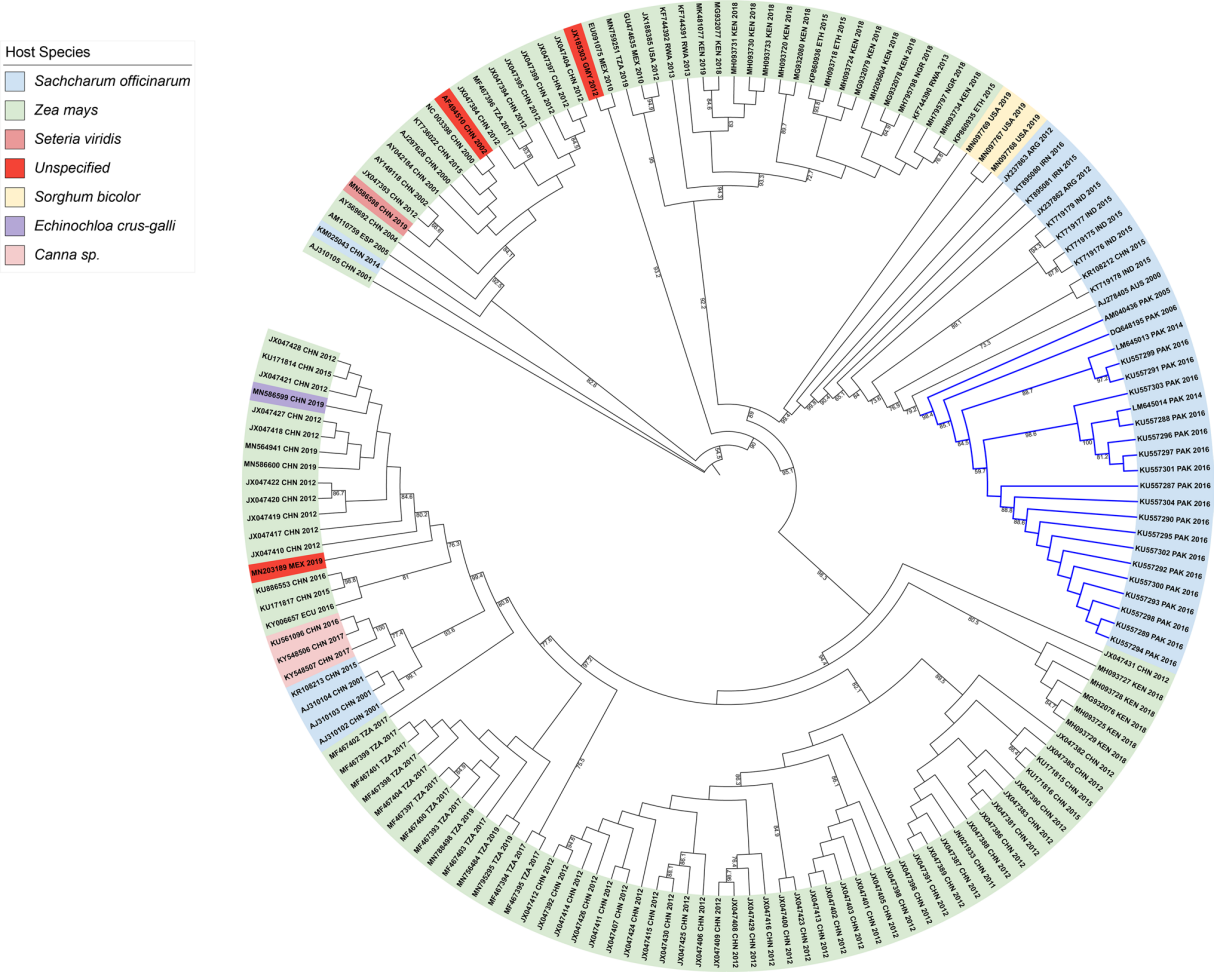
Phylogenetic analysis based on the CP nucleotide sequences, using the ML method. The tree consists of 161 CP sequences identified here by NCBI accession number, followed by the country of origin and date of NCBI entry. The colors indicate the host plant and are explained in the legend. The branches with sequences from Pakistan are shown in blue.

Previous studies reporting SCMV isolates from a single country and a common host have shown a low frequency of polymorphisms in the CP sequences, but polymorphisms were more frequent between isolates from different hosts [16, 17, 19, 36]. Since there are more reported CP sequences than full genome sequences for SCMV, we performed ML analysis to determine if CP sequences that cluster into phylogenetic subgroups are more likely to be defined by host than by geography, like the full-length genome sequences. We examined 161 SCMV CP nucleotide sequences from NCBI, including sequences from Pakistan, which was not represented in the full-length genome sequences (Supplementary Tables S1 and S3) [14, 18, 21]. The sequences ranged from 971 to 1119 nt in length, and the alignment was trimmed at both ends to restrict analysis to the conserved 539 nt, stretching from position 447 to 970 in the alignment. This region resembles the “coherently evolving coat protein” (cCP) region used for broad potyvirus phylogeny studies by Gibbs et al. [6]. This region is more likely to evolve by point mutations and less by recombination. The most apparent pattern within this ML phylogeny is the clustering of isolates that primarily share common hosts but occur in various locations in the world (Fig. 5). For example, the sugarcane-infecting viruses from Argentina, Iran, India, China, Australia, and Pakistan form a large subgroup, further highlighting the importance of the host plant species in determining the CP sequence.

The phylogeny also showed individual isolates that have a common country of origin but different hosts that are closely related (Fig. 5). For example, four SCMV isolates infecting *S. officinarum* (sugarcane), three infecting *Canna* spp., and several more infecting maize were collected in China and appear to be closely related although the sugarcane-infecting isolates were collected in an earlier decade from other host isolates.

## Discussion

SCMV is one of the most important cereal viruses globally and is among the most important diseases of sugarcane in tropical and subtropical areas. Sugarcane is used not only for sugar production but also as a source of biofuels through the production of cellulosic ethanol [18]. While the genetic diversity of SCMV has been analyzed by other investigators in prior studies, the number of available sequences of SCMV isolates remains limited [7, 14, 19, 20]. By including isolates from further geographic locations and adopting new bioinformatic tools to study complete genomes and CP sequences, we are beginning to understand the molecular evolution of SCMV. Given that the number of SCMV CP sequences in the NCBI database exceeds the number of available full genome sequences and represents a somewhat wider geographic distribution, this study was carried out to determine whether the available CP sequences from samples from Pakistan would provide sufficient information to assess their geographical or host provenance. Toward this goal, we first compiled and analyzed 139 complete SCMV genome sequences and identified four major groups that extend from three deeply rooted branches. The genome sequences diverged mostly according to host species as well as geographical region, and thus, SCMV sequences isolated from maize clustered separately from those recovered from canna, sorghum, or sugarcane on the same or different continents, although there were some individual isolates in these clusters that were likely recovered from weeds associated with the same agricultural fields. Similar observations have been reported in previous studies [7, 14, 16].

The strength of these rooted branches was valuable for investigating the phylogeography of SCMV. There were a few discordant isolates that were in the same subgroup with isolates from a different global region. These few events could be explained by the recent expansion (since 2018) in the global trade of cereal crops such as sorghum and maize (PSD Online (usda.gov)) [37], as described in economic reports from the Global Agricultural Information Network (https://gain.fas.usda.gov/). It is worth hypothesizing that the few isolates that are discordant reflect the movement of agriculture seed and products between major exporters and their customers in other countries [3].

The recombination events identified in this study represent events that may be closely related to their phylogeography, with a few examples representing distinct geographic regions. These latter examples could represent single recombination events that occurred in a specific locale and were then distributed to different regions by trade among countries. Understanding when and where the recombination events occurred is hampered by the small number of SCMV isolates sequenced that are spatially distributed, compared to the thousands of species and isolates of begomoviruses, for example, where a single species may have 700–1000 fully sequenced isolates, allowing for robust statistical investigations of recombination hotspots [38, 39].

In recombination analysis, the largest number of isolates had breakpoints in the NIb-CP region, followed by the 6K1-CI region, and then the 6K2-VPg-Pro region. Fewer breakpoints occurred within the P1, HC-Pro, or P3/PIPO regions [14, 16, 17, 19, 36]. It is interesting that the selection pressures on the CI, VPg, Pro, and NIb regions are constrained by their molecular roles in virus replication. In contrast, the overall CP sequence, and in particular the hypervariable region of the CP, is constrained by host interactions and aphid vectoring [15]. Thus, recombination events in the NIb-CP region can have multiple effects on virus replication, vascular transport, and vectoring.

Here, combined phylogenetic and recombination analysis was used as a reference for studying isolates for which only the CP sequences are available. We used 161 SCMV CP sequences, including ones from Pakistan. The ancestral sequences of SCMV CP isolated from sugarcane showed different branch lengths according to their origin in Pakistan, India, Iran, Australia, Argentina, and China. The sequences from Pakistan showed a high degree of similarity to those of Australian isolates. The phylogenetic lineages more often reflect the particular niche, in this case the host plant species.

In summary, we looked at the currently available complete genome sequences of SCMV isolates representing different hosts and geographical regions. We observed evidence of extensive recombination among different SCMV isolates. The clustering of SCMV isolates into groups was more often due to a common host than to geography. This research is limited because full-length SCMV genome sequences are available from only a few countries. To capture the spatiotemporal dynamics of SCMV, it is essential to have a global effort to collect and sequence SCMV genomes in the future.

## Electronic Supplementary Material

Below is the link to the electronic supplementary material

Supplementary Material 1 (XLSX 63 KB)

## Data Availability

All genome nucleotide and polyprotein sequences were obtained from the NCBI GenBank database. The curated genome sequences and accession numbers underlying this study are provided in Supplementary Table S1. Khalid Muhammad was sponsored by Higher Education Commission of Pakistan. Khadija Ahmed was sponsored by an HEC NRPU-5968 grant.
